# Supplementary material for: Pyruvate Dehydrogenase Kinase 1 inhibition mediated oxidative phosphorylation enhancement in cartilage promotes osteoarthritis progression
Source: BMC Musculoskelet Disord. 2023 Jul 20;24:597. doi: 10.1186/s12891-023-06585-6 (PMC10357736; doi:10.1186/s12891-023-06585-6)
Supplement: Supplementary file 1 — Additional file 1. [file 12891_2023_6585_MOESM1_ESM.zip › BP.Gsea.1653623667859/GOBP_MATURATION_OF_5_8S_RRNA_FROM_TRICISTRONIC_RRNA_TRANSCRIPT_SSU_RRNA_5_8S_RRNA_LSU_RRNA.html]

Details for gene set GOBP\_MATURATION\_OF\_5\_8S\_RRNA\_FROM\_TRICISTRONIC\_RRNA\_TRANSCRIPT\_SSU\_RRNA\_5\_8S\_RRNA\_LSU\_RRNA[GSEA]

|  || Dataset | 55457\_collapsed\_to\_symbols.cls.cls #OA\_versus\_normal.cls.cls #OA\_versus\_normal\_repos |
| Phenotype | cls.cls#OA\_versus\_normal\_repos |
| Upregulated in class | normal |
| GeneSet | GOBP\_MATURATION\_OF\_5\_8S\_RRNA\_FROM\_TRICISTRONIC\_RRNA\_TRANSCRIPT\_SSU\_RRNA\_5\_8S\_RRNA\_LSU\_RRNA |
| Enrichment Score (ES) | -0.56936276 |
| Normalized Enrichment Score (NES) | -1.7617373 |
| Nominal p-value | 0.0017152659 |
| FDR q-value | 0.11278072 |
| FWER p-Value | 0.998 |
Table: GSEA Results Summary

  

Fig 1: Enrichment plot: GOBP\_MATURATION\_OF\_5\_8S\_RRNA\_FROM\_TRICISTRONIC\_RRNA\_TRANSCRIPT\_SSU\_RRNA\_5\_8S\_RRNA\_LSU\_RRNA      
 Profile of the Running ES Score & Positions of GeneSet Members on the Rank Ordered List

  

| SYMBOL | TITLE | RANK IN GENE LIST | RANK METRIC SCORE | RUNNING ES | CORE ENRICHMENT || 1 | EXOSC7 | exosome component 7 [Source:HGNC Symbol;Acc:HGNC:28112] | 1969 | 0.364 | -0.1028 | No |
| 2 | ERI3 | ERI1 exoribonuclease family member 3 [Source:HGNC Symbol;Acc:HGNC:17276] | 4627 | 0.102 | -0.2951 | No |
| 3 | ABT1 | activator of basal transcription 1 [Source:HGNC Symbol;Acc:HGNC:17369] | 4783 | 0.088 | -0.2950 | No |
| 4 | RPP40 | ribonuclease P/MRP subunit p40 [Source:HGNC Symbol;Acc:HGNC:20992] | 8318 | -0.179 | -0.5447 | Yes |
| 5 | RPS21 | ribosomal protein S21 [Source:HGNC Symbol;Acc:HGNC:10409] | 8394 | -0.185 | -0.5251 | Yes |
| 6 | EXOSC10 | exosome component 10 [Source:HGNC Symbol;Acc:HGNC:9138] | 8404 | -0.186 | -0.5002 | Yes |
| 7 | WDR12 | WD repeat domain 12 [Source:HGNC Symbol;Acc:HGNC:14098] | 8505 | -0.194 | -0.4813 | Yes |
| 8 | EXOSC8 | exosome component 8 [Source:HGNC Symbol;Acc:HGNC:17035] | 8554 | -0.198 | -0.4578 | Yes |
| 9 | FTSJ3 | FtsJ RNA 2'-O-methyltransferase 3 [Source:HGNC Symbol;Acc:HGNC:17136] | 9193 | -0.247 | -0.4734 | Yes |
| 10 | RCL1 | RNA terminal phosphate cyclase like 1 [Source:HGNC Symbol;Acc:HGNC:17687] | 9586 | -0.282 | -0.4650 | Yes |
| 11 | URB1 | URB1 ribosome biogenesis homolog [Source:HGNC Symbol;Acc:HGNC:17344] | 9833 | -0.304 | -0.4422 | Yes |
| 12 | PES1 | pescadillo ribosomal biogenesis factor 1 [Source:HGNC Symbol;Acc:HGNC:8848] | 10268 | -0.347 | -0.4282 | Yes |
| 13 | NOL9 | nucleolar protein 9 [Source:HGNC Symbol;Acc:HGNC:26265] | 10361 | -0.357 | -0.3862 | Yes |
| 14 | EXOSC9 | exosome component 9 [Source:HGNC Symbol;Acc:HGNC:9137] | 10606 | -0.384 | -0.3523 | Yes |
| 15 | NOP14 | NOP14 nucleolar protein [Source:HGNC Symbol;Acc:HGNC:16821] | 10747 | -0.398 | -0.3084 | Yes |
| 16 | RRS1 | ribosome biogenesis regulator 1 homolog [Source:HGNC Symbol;Acc:HGNC:17083] | 11045 | -0.437 | -0.2714 | Yes |
| 17 | ERI2 | ERI1 exoribonuclease family member 2 [Source:HGNC Symbol;Acc:HGNC:30541] | 11320 | -0.474 | -0.2275 | Yes |
| 18 | UTP20 | UTP20 small subunit processome component [Source:HGNC Symbol;Acc:HGNC:17897] | 11656 | -0.526 | -0.1812 | Yes |
| 19 | KRI1 | KRI1 homolog [Source:HGNC Symbol;Acc:HGNC:25769] | 12074 | -0.613 | -0.1293 | Yes |
| 20 | EXOSC2 | exosome component 2 [Source:HGNC Symbol;Acc:HGNC:17097] | 12100 | -0.620 | -0.0459 | Yes |
| 21 | BOP1 | BOP1 ribosomal biogenesis factor [Source:HGNC Symbol;Acc:HGNC:15519] | 12580 | -0.785 | 0.0248 | Yes |
Table: GSEA details [plain text format]

  

Fig 2: GOBP\_MATURATION\_OF\_5\_8S\_RRNA\_FROM\_TRICISTRONIC\_RRNA\_TRANSCRIPT\_SSU\_RRNA\_5\_8S\_RRNA\_LSU\_RRNA      
 Blue-Pink O' Gram in the Space of the Analyzed GeneSet

  

Fig 3: GOBP\_MATURATION\_OF\_5\_8S\_RRNA\_FROM\_TRICISTRONIC\_RRNA\_TRANSCRIPT\_SSU\_RRNA\_5\_8S\_RRNA\_LSU\_RRNA: Random ES distribution      
 Gene set null distribution of ES for **GOBP\_MATURATION\_OF\_5\_8S\_RRNA\_FROM\_TRICISTRONIC\_RRNA\_TRANSCRIPT\_SSU\_RRNA\_5\_8S\_RRNA\_LSU\_RRNA**
